# Persistent hypokalemia in an acute lymphoblastic leukemia patient

**DOI:** 10.4103/0971-5851.64255

**Published:** 2009

**Authors:** Altaf Ali, Sheikh Ajaz Aziz, Afaq Khan, Sajad Gelani

**Affiliations:** *Department of General Medicine, Sher-I-Kashmir Institute of Medical Sciences, Srinagar, India*

**Keywords:** *Trans-tubular potassium gradient*, *venous blood gas*, *nephrotoxicity*

## Abstract

Nephrotoxicity is an important adverse effect of Amphotericin B, and although risk factors for nephrotoxicity in adults have been investigated, studies examining nephrotoxicity in the pediatric population are scarce. We describe case of 10 year old boy (CALLA – VE B cell ALL) who received conventional Amphotericin-B, but he persisted with hypokalemia.

## CASE REPORT

10 year old boy who was diagnosed as a case of CALLA – VE B Cell ALL (Acute lymphoblastic leukemia) was started on UKALL XII PROTOCOL (drugs that were part of protocol were Prednisolone, Vincristine, Danorubicin, L-Asparagine, 6-MP, Cytarabine, and Cyclophosphamide). He received the first induction uneventfully. During the second induction the patient developed febrile neutropenia and was treated with Cefipime, Amikacin and vancomycin. There was apparently no focus and the cultures were sterile, seeing no response the patient was started on conventional Amphotericin-B, his baseline kidney function tests function tests were (Urea-24,Creatinine-0.4) and (serum potassium-4.2). During treatment, the patient developed severe hypokalemia (Sr-K; 2.1, 2.6 mEq/L) and was started on IV replacement of potassium along with IV replacement of magnesium. The patient received Amphotericin-B for almost 40 days (Total cumulative dose 1 gm). The patient responded and Amphotericin was stopped, with serum potassium at discharge being 3.8 mEq/L).

The patient was again readmitted after one month and his investigations showed Serum creatinine - 0.45 mg/dl. VBG and electrolytes-Ph : 7.41, Hco_3:_26, serum potassium : 2.1 mEq/L (repeat tests same), (serum magnesium-2.8), (Urine examination : pus cells 3 - 4,albumin / sugar - nil), (24 hour urinary volume : 1.5 liter). Assuming that some damage to renal tubules must have occurred due to Amphotericin, Hypokalemia was evaluated (Urine k-20 mEq/L), (Urine sodium-134 mEq/L (undiluted)), (Serum osmolality-287 mosm/L), (Urine osmolality- 400 mEq/L), and TTKG-13, indicating ongoing renal loss. Presently the patient is on both magnesium and potassium replacement, but despite that, the serum potassium is low all along.

## DISCUSSION

It has been clearly documented that Amphotericin-B induces renal potassium wasting and can produce substantial potassium deficit. Levels below 3 mmol/L have been reported in 12 to 40% of the patients in recent publications.[1–4] It has been proposed that both tubular injury and renal vasoconstriction play an important role in Amphotericin-B nephrotoxicity.[5,6] Our patient did not develop this complication because of the chemotherapeutic agent or antibiotic therapy, as persistent hypokalemia and mild metabolic acidosis is caused only by Amphotericin B. Our case clearly documents the fact that even after Amphotericin-B was stopped the patient could continue with hypokalemia, which might be a single indicator of tubular damage.

## References

[1] Goldman RD, Koren G (2004). Amphotericin B nephrotoxicity in children. J Pediatr Hematol Oncol.

[2] White MH, Bowden RA, Sandler ES, Graham ML, Noskin GA, Wingard JR (1998). Randomized, double-blind clinical trial of amphoterin B colloidal dispersion vs.amphotericin B in the empirical treatment of fever and neutropenia. Clin Infect Dis.

[3] Walsh TJ, Finberg RW, Arndt C, Hiemenz J, Schwartz C, Bodensteiner D (1999). Liposomal amphotericin B for empirical therapy in patients with persistent fever and neutropenia. N Engl J Med.

[4] Prentice HG, Hann IM, Herbrecht R, Aoun M, Kvaloy S, Catovsky D (1997). A randomized comparison of liposomal versus conventional amphotericin B for the treatment of pyrexia of unknown origin in neutropenic patients. Br J Haematol.

[5] Sawaya PB, Briggs JP, Schnermann J (1995). Amphotericin B nephrotoxicity: the adverse consequences of altered membrane properties. J Am Soc Nephrol.

[6] Tonomura Y, Yamamoto E, Kondo C, Itoh A, Tsuchiya N, Uehara T (2009). Amphotericin B-induced nephrotoxicity: characterization of blood and urinary biochemistry and renal morphology in mice. Hum Exp Toxicol.

